# Genome properties in 2019: a new companion database to InterPro for the inference of complete functional attributes

**DOI:** 10.1093/nar/gky1013

**Published:** 2018-10-26

**Authors:** Lorna J Richardson, Neil D Rawlings, Gustavo A Salazar, Alexandre Almeida, David R Haft, Gregory Ducq, Granger G Sutton, Robert D Finn

**Affiliations:** 1 European Molecular Biology Laboratory, European Bioinformatics Institute (EMBL-EBI), Wellcome Genome Campus, Hinxton, Cambridge CB10 1SD, UK; 2 J. Craig Venter Institute (JCVI), 9605 Medical Center Drive, Suite 150, Rockville, MD 20850, USA

## Abstract

Automatic annotation of protein function is routinely applied to newly sequenced genomes. While this provides a fine-grained view of an organism's functional protein repertoire, proteins, more commonly function in a coordinated manner, such as in pathways or multimeric complexes. Genome Properties (GPs) define such functional entities as a series of steps, originally described by either TIGRFAMs or Pfam entries. To increase the scope of coverage, we have migrated GPs to function as a companion resource utilizing InterPro entries. Having introduced GPs-specific versioned releases, we provide software and data via a GitHub repository, and have developed a new web interface to GPs (available at https://www.ebi.ac.uk/interpro/genomeproperties). In addition to exploring each of the 1286 GPs, the website contains GPs pre-calculated for a representative set of proteomes; these results can be used to profile GPs phylogenetically via an interactive viewer. Users can upload novel data to the viewer for comparison with the pre-calculated results. Over the last year, we have added ∼700 new GPs, increasing the coverage of eukaryotic systems, as well as increasing general coverage through automatic generation of GPs from related resources. All data are freely available via the website and the GitHub repository.

## INTRODUCTION

Modern DNA sequencing technologies have revolutionized our ability to sequence DNA for not only isolate organisms, but also collections of organisms (metagenomics). While the automatic transfer of annotations from a handful of characterized sequences to the genes encoded in a novel genome may be considered somewhat routine, especially for prokaryotic genomes, it nonetheless requires the identification of functional data in the scientific literature, as well as a method of defining those sequences that should acquire the transferred annotation. For the majority of automatic annotation in UniProtKB (1), the comprehensive protein sequence knowledgebase, those sequences are identified by InterPro (2) using profile-based protein family models (such as position specific scoring matrices (PSSMs) or profile hidden Markov models (HMMs)), provided by various protein families databases and integrated into InterPro. These models provide much greater sensitivity in detecting diverse protein family members in comparison to single sequence matching methods.

While the annotation of individual genes and proteins is an important prerequisite to understanding how an organism is adapted to its ecological niche, higher order functions are more often than not performed by multiple proteins. For example, where multiple proteins come together to form a functional complex, such as a transporter system, or where multiple proteins are required in a pathway, such as the biosynthesis of proline from glutamate, which is a four-step process requiring three different enzymes to catalyse three steps in the pathway. Resources such as the Kyoto Encyclopedia of Genes and Genomes (KEGG) (3) and SEED subsystems (4) have been used extensively for the annotation of pathways, complexes and networks. While KEGG is widely used, certain parts of the data are no longer free for users, thus restricting use. Both KEGG and the SEED subsystems rely on BLAST-based searches for the transfer of genome annotations (5, 6). When first introduced, these BLAST-based methods had superior speed compared with HMMER2-based profile HMMs used by protein family databases. However, as the number of sequenced genomes has increased over time, the size of both reference and target sequence databases have significantly increased. This has a negative impact on the speed of pairwise BLAST-based searches, and has led to the adoption of algorithms which implement heuristics (e.g. GHOSTX (7)) to improve search speed. The advent of HMMER3 (8), along with more recent iterative improvements have enhanced the speed of profile HMM-based searches such that they are now equivalent to those of BLAST. As profile-based protein family reference databases are much smaller, and grow at linear rates whilst maintaining coverage, they offer a scalable and more sensitive solution compared to single sequence-based searches. This sensitivity is particularly important in relation to metagenomics where the analysis includes diverse organisms that are not reflected in the reference database (9,10).

Genome Properties (GPs) was originally developed as an extension to the TIGRFAMs resource, providing a method to improve the functional annotation of prokaryotic genomes, and assist in comparative genomics (11–13). In essence, it consists of a queryable set of molecular reconstructions (e.g. of pathways), which allow the inference of the higher order functions that may be encoded in any given genome. For example, an organism can be proposed to synthesize biotin if its genome can be shown to encode the complete set of proteins required to perform the relevant biochemical steps in the pathway. The previous versions of GPs primarily utilized the profile HMMs produced by TIGRFAMs, which were supplemented by a small subset of Pfam (14) profile HMMs to determine the presence of the required proteins in the property. Restricting the available models to just these resources meant that there was a limitation in the number of specific family models available for use, as well as the taxonomic range of organisms that were able to be annotated.

TIGRFAMs and Pfam are both part of InterPro, a freely available resource that allows users to classify protein sequences into families and predict important domains and sites within protein sequences (2). The breadth and depth of annotation in InterPro is achieved by combining protein family and domain prediction models (including, but not restricted to, profile HMMs) from a consortium of 14 specialist member data resources. The various protein models are combined to produce InterPro entries describing each protein family, domain or site in a unified way. InterProScan (15) is the software that underpins the comparison of protein sequences against the InterPro predictive models. InterPro matches for all protein sequences contained in the UniProtKB resource (1) are calculated on a monthly basis, providing a comprehensive and up-to-date set of functional annotations for all UniProtKB sequences.

In light of the significantly larger collection of protein families models available in InterPro, we have extended GPs such that any InterPro entry (and hence associated member database signature) can be used to represent a GPs step. GPs has been migrated to EMBL-EBI and is now a companion database to InterPro. This allows GPs to leverage the InterProScan calculations that already exist for all UniProtKB sequences, thereby offering a simple and efficient process to predict the existence of a GP for any given species for which a proteome exists in UniProtKB. Herein, we describe the numerous developments to GPs during the transition to using InterPro, the functionality of the new GPs website, and the expansion in number of available GPs.

## GENOME PROPERTIES DATA AND CALCULATION

### Genome properties entry file format

The original GPs dataset (as hosted at J. Craig Venter Institute (JCVI)) was stored in a Sybase relational database, which lacked any form of external or portable curation interface. To simplify the data structure and curation of GPs, a flatfile format (*DESCription file*; DESC file) was designed that fully described each individual property. The format of the DESC file closely follows that of both the TIGRFAMs and Pfam DESC files, producing files in a format familiar to users and curators. A detailed description of the DESC file format is provided in the GPs documentation (https://genome-properties.readthedocs.io/en/latest/index.html). Briefly, each DESC file is divided into two parts: the top half describes the property as a whole, including a one-line description of the property, a free-text field describing the property, as well as appropriate cross references to literature and other databases. In addition to these fields, there is a *type* field. There are currently six *types* of GP: pathway, metapath, system, guild, complex and category. The first five designate the various classes of functional attributes being described in each case. Categories are distinct from the others in that they do not seek to model a particular functional system but rather exist as organizational properties, allowing the other GPs to be viewed as a hierarchy. ‘Complex’ is a new type that we have added to the original five GP types, introduced to represent known macromolecular complexes (described in further detail below).

The second half of the DESC file contains the molecular reconstruction (as a series of steps) that constitute that GP. Each step has at least one line of evidence to determine its presence within the proteome being analysed. This evidence can be one of two classes: (i) another GP (found in metapaths and categories) or (ii) an InterPro entry. Each step can be flagged as either *required* (necessary for the function of the property being modelled) or otherwise deemed *optional.* An important step in the integration of GPs to InterPro was the assignment of InterPro accessions to steps. For the majority of entries, the original evidence using TIGRFAMs and Pfam models have simply been uplifted to their corresponding InterPro entry. In cases where those original TIGRFAMs and Pfam models were not currently integrated into an InterPro entry, some curatorial input was required. While the ‘missing evidence’ was integrated wherever possible, another existing InterPro entry and model was chosen as replacement evidence for the step in cases where this wasn’t feasible.

Further to the DESC file, a GP may also have a FASTA file that contains at least one example sequence that will resolve to a ‘yes’ for each step represented by an InterPro entry. These were created primarily for internal validation purposes as InterPro entries (and the constituent member databases) are not static, and as such, the chosen evidence may necessarily have to change over time. These FASTA files represent static examples of each protein being modelled within a GP, that should always be a match for any evidence used to define that step. Finally, each property also has a status file specifying if the property has been checked and can be made available in the GPs release/website (both described below). These files are collected within a single containing folder that represents a GPs entry.

The original ‘TIGRFAMs’ GPs dataset contained 1077 properties, of which only a subset (584) was integrated into the InterPro companion resource. As well as those properties which conform to the description above, the ‘TIGRFAMs’ GPs set also included properties that utilize various other metrics to determine their presence/absence in a genome. For example, a substantial subset relies on information about the relative location of genes within the genome coding for the step evidence proteins. While such information is readily available in the context of analysing a genome, it is essentially lost when analysing a proteome as described here. It is clear that the inclusion of such information on genomic context can provide increased confidence in some GPs results, for example through determining if genes encoding individual steps exist within the same operon. However, while this is beyond the current scope of our reimplementation, it remains an area of focus we hope to pursue in future releases, given the potential benefits to validating results. Further subsets of the original ‘TIGRFAMs’ GPs rely on other calculable properties (such as GC content) or on manual assignment of relevant species. These other metrics are also beyond the scope, and as such these properties were not able to be included in the initial migration.

### Accessing, contribution and validating GP entries

In an effort to enable GPs to be both fully accessible and freely available, all our data and software associated with the curation, release and calculation are stored in a GitHub repository (https://github.com/ebi-pf-team/genome-properties). In addition to facilitating access to the data (i.e. the DESC files), it allows users to report issues, suggest improvements to a property, and even propose new properties. This can be accomplished either by generating an issue on the repository website, using the GPs helpdesk email (GenProp@ebi.ac.uk) or by modifying a local checkout and generating a pull request. Indeed, the GitHub repository provides a full revision history of any changes that have been made to a particular property and by whom. Finally, the GitHub repository also contains basic documentation and downloadable bundles for each release version (discussed in further detail below).

### Asserting the presence of a GP

For the GP result to be a *yes* for any particular species, proteins matching the evidence for all required steps in that property must be found within the proteome of the species in question, asserted by being present in a single input file. In reality, there a number of reasons why it may not be possible to match evidence for every step. Some protein families are challenging to model, leading to a lack of existing suitable protein signature models to use as evidence. Alternatively, the specific enzyme required to carry out a step within a pathway may not be known. Equally, a particular step may be performed by a protein family that matches multiple proteins. While in a genome it may be feasible to ensure that the genes belong to a single operon, this is not possible when dealing with a set of proteins that come from a genome. Therefore, each property is assigned a threshold value to factor in such cases. This specifies the number of matched steps, above which a partial result can be reported, which is considered as a positive result. If the number of matched steps falls on or below the threshold, the property is reported as ‘not found’ in that species. These threshold values are not defined automatically (except in the case of semi-automatically produced GPs, described below) but rather they are manually curated on a property by property basis. In addition to accommodating the example scenarios detailed above, this curation of threshold values also allows for step evidences that function in multiple GPs to be taken into account, as they should not be considered as evidence of a property without supporting steps.

The aforementioned GPs GitHub repository includes the software for assigning the properties (*assign_genome_properties.pl*), whose use is described in more detail on the *calculating genome properties* page of the website (https://www.ebi.ac.uk/interpro/genomeproperties/#calculating). The script has a variety of output options with differing levels of detail, from summarizing which GPs are found, partial or absent, to a longer form that includes the result for each step, as well as a protein-centric report, indicating which proteins matched the evidence for a particular step. This script can be downloaded to analyse collections of proteomes locally. In addition to assigning GPs included in the latest release, the software is agnostic about the origin and content of the GPs file (genomeProperties.txt). This feature allows us (and users) to experiment with the development of new properties, before they are submitted to the resource by editing the file to include the experimental GP.

## EXPANDING GENOME PROPERTIES

The increased scope of InterPro entries has now allowed the addition of 702 new GPs (numbers correct for GP v2.0.1) since the original migration. A proportion of these new GPs (∼70) have been manually generated, although the manual creation of GPs is a time-consuming and curation-heavy process. In an effort to increase the coverage of GPs in a timelier fashion, we pursued a process of integrating data from other macromolecular complex and pathway resources, to semi-automatically generate GPs describing entities found in those resources, thereby minimizing the amount of curation required.

### Genome properties of type COMPLEX

One source of semi-automatically generated GPs came from the Complex Portal (16), a resource that describes macromolecular complexes. The physical molecular interaction data found in the Complex Portal is derived from the literature and is hence restricted to a few key model organisms. In this first import from the Complex Portal, we focused on macromolecular complexes found in *Escherichia coli*, with a view to using GPs to identify the corresponding complexes in related organisms. As the Complex Portal provides cross-references to both InterPro and UniProtKB, we automatically extracted the data to generate draft GPs entries. In the majority of cases, the UniProtKB sequence would match multiple InterPro entries, consequently a minimal curation step was included to select which InterPro entry should be used as evidence in each case. Typically, InterPro entries of type *family* were chosen preferentially over other entry types such as *domain* or *homologous superfamily* as they tend to be more specific. A single protein can also match multiple InterPro entries of the same type within a hierarchy, therefore in each case the most specific InterPro entry (lowest in the hierarchy), which still provided broad taxonomic coverage, was chosen. While the taxonomic range of each InterPro entry was not specifically compared, the consistent approach to their selection was aimed at being conservative. If a homologue for each complex component exists in a particular species, there is a strong likelihood that the complex will be found in that particular organism.

### Additional PATHWAY genome properties

MetaCyc (17) is a database of highly curated metabolic pathways covering all domains of life. Each MetaCyc entry is specific for a defined species, or set of species. While these data are highly curated, the MetaCyc website does not provide any form of sequence-similarity annotation transfer system, and therefore cannot be used to infer pathway function in species outside of those specifically covered. Through collaboration with MetaCyc and the TIGRFAMs team at JCVI, we performed an analysis of the data within MetaCyc to establish the subset of pathways that define a particular protein for every reaction within the pathway. This allowed us to automatically assign matching InterPro entries for each reaction/step, and so generate GPs automatically. A second pass was then carried out to filter the matched InterPro entries. This automatic filter used the same criteria as the manual selection described for Complex Portal entries, namely preferentially selecting InterPro type ‘family’ entries, and then further selecting the more specific entries. It should be noted that this is an entirely uncurated process, and as such, these data should be treated with more caution as specified in the description of these properties on the GPs website. However, this automated process allowed a substantial expansion of the coverage by GPs. It was never the goal to recreate the content of the MetaCyc resource at GPs, and we do not include any pathway descriptions for these GPs; rather we provide links back to MetaCyc for users to retrieve the pathway information there. Including these data in GPs allows assertions of these MetaCyc pathways to be made automatically for novel genomes/proteomes that would otherwise be challenging to achieve.

### New eukaryote-specific genome properties

The original GPs were biased towards prokaryotes, which is unsurprising considering TIGRFAMs - a prokaryotic protein family database - was primarily used for the evidence in these properties. Utilizing relevant InterPro entries, we have now expanded GPs to include eukaryote-specific properties, using MEROPS (18) as a source of new properties. The MEROPS website is a resource of proteolytic enzymes, and includes annotation of pathways that include these enzymes (and cross references KEGG pathways). Typically, while a very specific protein family annotation is required for identification of a proteolytic enzyme, most entries in InterPro that only use a profile HMM (e.g. Pfam) include homologous members with different specificities. PANTHER (19), another InterPro member database, uses a competitive scoring post-processing system to differentiate between related PANTHER subfamilies. Therefore, for the generation of MEROPS-sourced GPs, the evidences used were mostly PANTHER subfamilies, as they provided the necessary level of specificity of evidence.

A consequence of the generation of new GPs from each of the resources described above was the requirement for some new type *category* properties to contain them, as the existing categories were designed to group predominantly prokaryotic properties. A total of 18 new categories were generated loosely based on grouping terms available at each of the source resources. The Complex Portal contains GO annotations (20) for most entries, therefore we were able to use high-level GO term assignments as a basis for category terms for Complex Portal-based entries. MetaCyc, on the other hand, already groups the pathways it describes into a hierarchy. This allowed us to use a subset of the ‘superclass’ terms provided by MetaCyc as a basis for the categories needed to describe MetaCyc-based GPs.

## GENOME PROPERTIES RELEASES AND WEBSITE

### Genome properties releases

Prior to the migration, GPs releases had been tied to the TIGRFAMs release cycle. A significant alteration since migrating to InterPro is the establishment of independent GPs release versioning. While the GPs release cycle has been decoupled from the InterPro release cycle, each GPs release is tagged with the relevant InterProScan version upon which the GPs entries are built. The initial GPs release within InterPro (containing the subset of ‘TIGRFAMs’ GPs data described previously) became v1.0, the most recent release at time of writing is v2.0.1 (InterProScan version 5.30–69.0). Release notes are provided detailing not only the InterProScan version requirement, but also the counts of new properties of each type, as well as major sources of new data within that release.

Various quality control checks are carried out on the data as part of the release process. These include ensuring that all properties are connected to the organizational hierarchy, either as members of a category, or as an evidence within a metapath. This ensures a path to every property within the browse feature of the website (see below). Further checks are carried out to verify that all InterPro IDs used as evidence are valid for the relevant InterProScan release, and that all properties requiring a FASTA file have one in place. A number of flatfiles (available within the GitHub repository) are produced as part of the release process, including a concatenation of DESC files for all properties included in the release. This file (genomeProperties.txt) provides an easy format for parsing, allowing release-specific GPs data to be used in external tools and workflows. Finally, a representative set of genomes/proteomes was established, which match a broad range of different GPs, and for which GPs matches/results are calculated at each release. These results are used on the website for the visualization of GPs data.

### Website production and design of viewer

The GPs website, https://www.ebi.ac.uk/interpro/genomeproperties, is the predominant method by which we anticipate users will interact with GPs. The site can be broadly divided into three main sections: Browse, Viewer and About.

**Figure 1.**
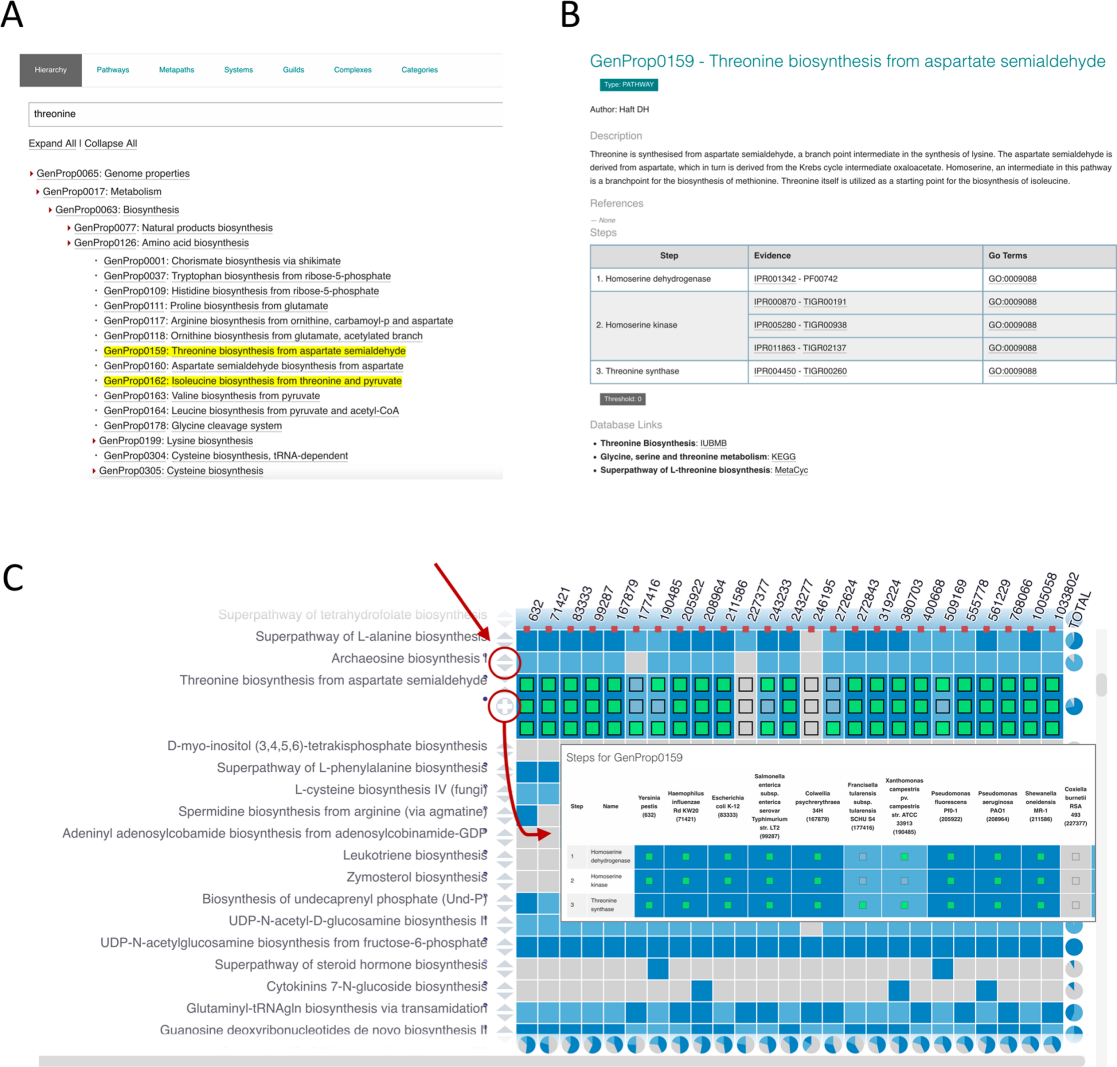
(**A**) Browse feature of website, showing a search for the term ‘threonine’ with the resulting matches within the hierarchy highlighted in yellow. (**B**) Example entry page showing contents of the DESC file for GenProp0159 - Threonine biosynthesis from aspartate semialdehyde. (**C**) Website viewer loaded with data for gammaproteobacteria (taxonomy IDs of species shown along the top), and GPs terms filtered for ‘biosynthesis’. Colours of cells indicate the result for each GP in each species (dark blue = yes, light blue = partial, grey = no). Clicking on the expansion arrows (indicated by the top red circle and arrow in figure) opens up the step results for that GP, as shown for Threonine biosynthesis from aspartate semialdehyde. Clicking on the + sign which is revealed (indicated by the bottom red circle in figure), opens a pop-up (indicated by the bottom red arrow in the figure) providing the step names as well as step results for that GP.

The *Browse* section provides lists of properties categorized by type, as well as a hierarchy of properties that can be browsed (Figure 1A). A useful feature is the search functionality available for the hierarchical tree. This performs a simple string search on the property names. Having identified the property of interest, selecting it opens the property page showing the contents of the DESC file for that property in a human readable format (Figure 1B).

The *Viewer* (Figure 1C) assists in the visual exploration of the representative proteomes, allowing the user to focus on species or pathways of interest. Furthermore, it permits the comparison of user-defined proteomes with these representative proteome sets. The viewer is organized as a matrix, with columns representing species (arranged as a taxonomic tree), and rows representing properties. The colour of each cell in the matrix indicates the presence (dark blue) or absence (grey) of the GP in the species. A lighter blue indicates partial evidence of the presence of the GP. To allow full interpretation of such partial matches, each row in the matrix can be further expanded to display the results for each individual step within a property. Users can specify the representative species that they would like to see the data for, either by navigating the taxonomic tree, or by searching with species name or tax ID. It is also possible to upload a novel user-defined proteome for comparison against the representative set. The supported file format is the InterProScan TSV output file. GP results are calculated for the given file, and the results are displayed in the viewer alongside the existing set. Several filters and visualization options are available to limit the size and complexity of the resulting matrix.

Finally, the *About* pages provide background information on the concepts of GPs, as well as links to supporting resources such as documentation, stored using Read the Docs (https://genome-properties.readthedocs.io/en/latest/index.html), and online training modules. There are two supporting training modules, both hosted as part of the EMBL-EBI Train Online courses: a quick tour providing a brief overview of the website, and a tutorial covering more in-depth functionalities of the website as well as the underlying concepts of GPs.

## COVERAGE OF PROTEOMES

**Figure 2.**
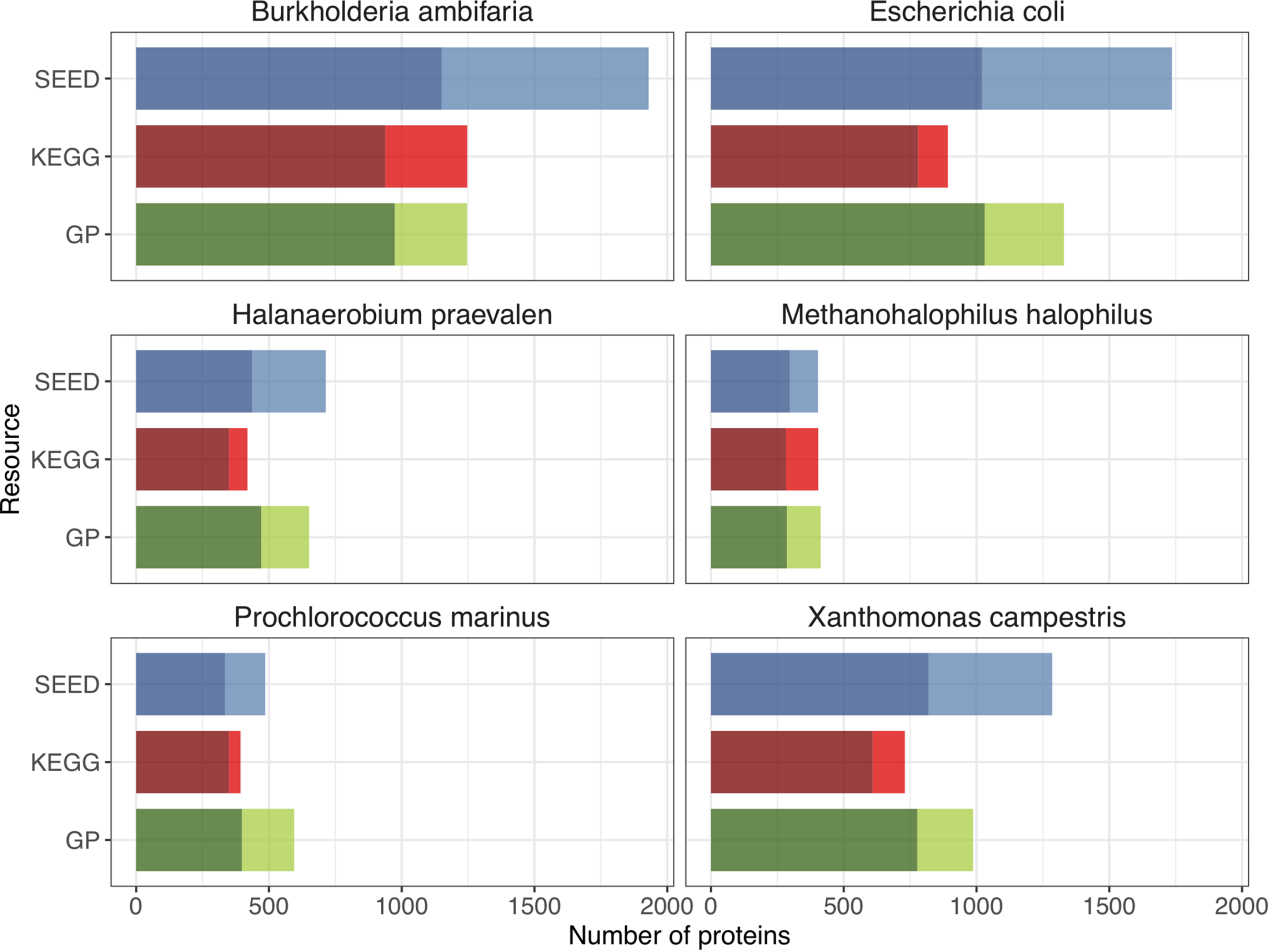
Comparison of KEGG, SEED subsystems and Genome Properties coverage of six different proteomes. Columns are coloured according to the three different resources. Darker shading within the column represents the amount of overlapping coverage (where a sequence is annotated by more than one resource), lighter shading represents unique coverage (sequences uniquely annotated by the resource). Note, in this figure only the species names are shown; specific strain information is provided in Table 1. Total proteome size (total number of sequences) for each species is also detailed in Table 1.

In an effort to understand the utility of GPs compared to other similar resources, namely KEGG and SEED subsystems, we were keen to undertake a comparison of the resources to quantify the overlap versus uniqueness of each resource. However, performing a meaningful comparison between these resources is not straightforward, as it is not possible to map directly between the resources due to the differences in granularity of entries, and in nomenclature used to describe similar or equivalent steps. Thus, we undertook to compare the number of sequences annotated by each resource for a small range of proteomes. These proteomes were chosen based on taxonomic range, as well as offering a range of proteome sizes and a distribution of well-studied model organism genomes to those that are relatively less experimentally characterized. To accomplish this, we downloaded the proteomes, SEED subsystems and KEGG pathway annotations from PATRIC (version 3.5.21) (21) for six different species. We calculated InterPro matches using InterProScan, thus determining the GPs results for each proteome based on the InterPro matches. To enable a fair comparison and eliminate the few non-specific models utilized by GPs which had the potential to over-inflate the number of sequence matches for GPs, we excluded all protein matches where a single step evidence matched more than three different proteins within the proteome. Figure 2 shows the overall annotation of each proteome by KEGG, SEED subsystems and GPs. Within each column, the two different shades indicate sequences that are annotated by one or more of the other resources, and sequences that are uniquely annotated by that resource. A more detailed breakdown of the coverage is provided in Table 1. All three resources provide a notable number of unique annotations for the six proteomes presented. Due to the broader scope of SEED subsystems and GPs, it is unsurprising that these always annotate more sequences than KEGG, and typically have the most annotations in common when comparing the resources pairwise. In two of the six proteomes (*Prochlorococcus marinus* and *Methanohalophilus halophilus*), GPs provides the greatest annotation coverage.

**Table 1.** Break-down of overlapping coverage between the different resources for six different proteomes, see text for details

Sequences annotated by:	ALL	GP & SS	SS & KEGG	KEGG & GP	KEGG only	SS only	GP only	Unannotated sequences	Total sequences
*Prochlorococcus marinus subsp. pastoris str. CCMP1986*	208	88	39	102	44	151	197	541	1370
*Methanohalophilus halophilus strain Z-7982*	151	75	71	60	122	106	127	1245	1957
*Escherichia coli K-12 strain K-12 C3026*	498	387	136	146	112	715	298	2257	4549
*Burkholderia ambifaria AMMD*	496	345	309	133	309	780	272	4537	7181
*Halanaerobium praevalens DSM 2228*	216	171	49	84	70	278	180	1083	2131
*Xanthomonas campestris pv. campestris str. B100*	381	303	135	93	121	466	210	2817	4526

## APPLICATION TO MICROBIAL GENOMICS

**Figure 3.**
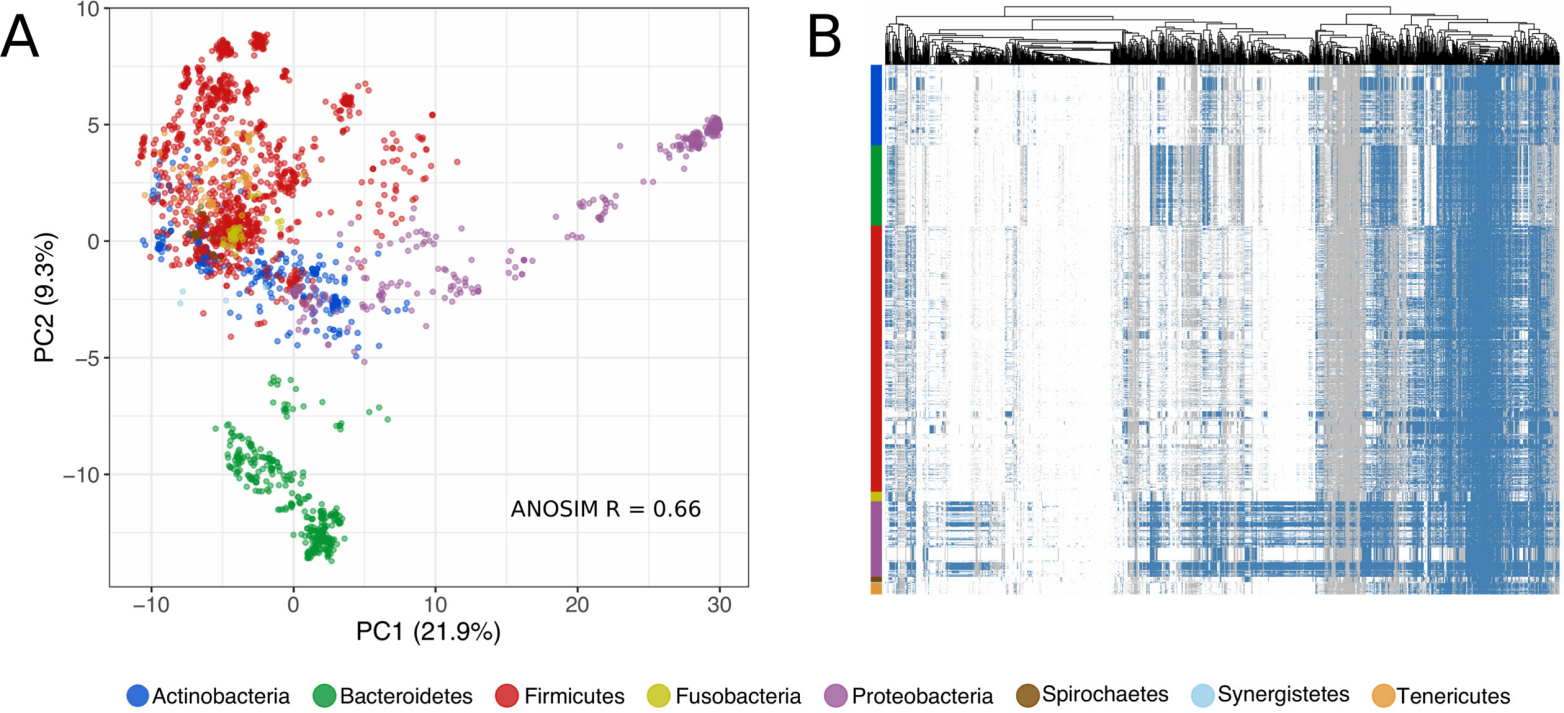
(**A**) Distribution of Genome Properties (GPs) of 2468 human-isolated genomes by Principal Component Analysis, with each point representing a genome and coloured by phylum. An ANOSIM test was performed by phylum using the Gower distance measure. (**B**) Heatmap depicting presence (blue), partial (grey) and absence (white) of the GPs (columns) contained within the human-specific genomes (rows). The dendrogram represents the hierarchical clustering of the GPs based on the patterns across the genomes.

GPs provides a useful annotation scheme to investigate the functional repertoire contained within complex microbial communities, primarily those from which complete protein sequences can be predicted and grouped together (e.g. genome/metagenome assemblies). To illustrate this, we investigated the GP profiles of a set of 2468 genomes from the Human Microbiome Project catalog (https://www.hmpdacc.org/catalog/) and the Human Gastrointestinal Bacteria Genome Collection (22). Protein coding sequences were first predicted for each genome using prodigal (23) and subsequently characterized with InterProScan (15). Thereafter, GPs results were calculated using the *assign_genome_properties.pl* script (available within the GitHub repository) on the InterProScan results. After converting the GPs results to numeric values (No = 0; Partial = 1; Yes = 2) we performed a Principal Component Analysis (PCA) of all these human-isolated genomes using the GPs to look for distinguishing patterns. This revealed a strong separation of the genomes according to their classified phylum (Figure 3A, ANOSIM *R* = 0.66, *P* < 0.001), highlighting that GPs are able to strongly differentiate the functional properties of genomes from distinct genetic backgrounds. Our results raise the possibility of leveraging GPs to complement and validate other taxonomy-based analysis types, e.g. derived from phylogenetic inference based on conserved marker genes (24, 25), which is vital for analysis of metagenomic assemblies. By further exploring the distribution of GPs across phyla (Figure 3B), patterns of presence/absence could be observed, where certain GPs were found exclusively in specific phylogenetic groups (e.g. Bacteroidetes), while others were conserved across all species. This exemplifies a useful strategy to identify core functions that may play a crucial role in adaptation to specific host environments, and can be extended to both reference and metagenome-assembled genomes. As more sequence data becomes available, having access to a reference catalogue of properties from particular biomes will provide important insights into the functional diversity and ecology of different microbial ecosystems.

## DISCUSSION

The integration of GPs into InterPro brings numerous benefits to both resources. While the protein signatures used to define the steps in the original GPs came from the TIGRFAMs and Pfam resources, InterPro has a further 12 member databases, whose protein signature models are integrated into InterPro entries. This greatly increases the potential pool of models, as well as taxonomic range, that can be used to define existing properties, as well as to create new properties.

One of the most significant benefits of using InterPro entries in the description of GPs is the ability to calculate GPs easily and swiftly, based on InterProScan results (which are maintained for UniProtKB). The coupling of such workflows allows users to efficiently analyse proteins encoded by genomes and metagenomes and thus make high level functional inferences, as well as being able to access those finer-grained details provided by InterPro and InterPro2GO. Calculating the output of all GPs for any given species, allows the resultant set of *present, absent* and *partial* results to be thought of as a fingerprint for that species. Comparing GPs fingerprints for a large number of genomes greatly reduces the computational overhead of phylogenetically profiling that set of genomes. In the case of bacterial genomes, rather than comparing the presence or absence of a few thousand protein families, users are able to compare the fingerprints composed of a few hundred GPs. Furthermore, as GPs provide a clear provenance of their calculation, such profiling approaches will allow the identification of taxonomic clades where a protein family in InterPro lacks sensitivity, or where an alternative step may have evolved.

In analysing the comparison of coverage between GPs, KEGG and SEED subsystems, it is unsurprising that there is a common core set of proteins annotated by all. Due to our import from MetaCyc, we have not included a comparison of GPs to MetaCyc, but as demonstrated here and previously (26), despite the common core set, KEGG, SEED subsystems, MetaCyc and GPs each have unique coverage due to the differences in scope of each of the resources and the functional pathways and systems that have been included to date.

Finally, the new close relationship of GPs with InterPro and UniProtKB creates opportunities for rapidly expanding GPs based on the annotations found in these resources, as well as the integration of further data, such as the Enzyme Commission (EC) numbers and reaction identifiers contained in Rhea and/or MetaCyc.
